# Determination of Oseltamivir Quality by Colorimetric and Liquid Chromatographic Methods

**DOI:** 10.3201/eid1404.061199

**Published:** 2008-04

**Authors:** Michael D. Green, Henry Nettey, Robert A. Wirtz

**Affiliations:** * Centers for Disease Control and Prevention, Atlanta, Georgia, USA

**Keywords:** Oseltamivir, bird flu, avian influenza, colorimetric, chromatography, research

## Abstract

We developed a colorimetric and chromatographic assay for oseltamivir to assess the authenticity of Tamiflu (F. Hoffmann-La Roche Ltd., Basel, Switzerland) because of a growing concern about counterfeit oseltamivir. The colorimetric assay is quantitative and relies on an extractable colored ion-pair complex of oseltamivir with Congo red or bromochlorophenol blue. The reverse-phase chromatographic assay uses an alkaline mobile phase with UV detection. Both methods were evaluated for variability and selectivity and subsequently applied to batches of oseltamivir products acquired through the Internet. The Congo red test showed greater assay sensitivity, linearity, and accuracy. Colorimetric and chromatographic analysis showed all batches of oseltamivir product were within ±15% of the stated amount of active ingredient.

## Abstract 2

The high performance liquid chromatography method is useful.

The antiviral drug oseltamivir phosphate has been recommended by Centers for Disease Control and Prevention as an adjunct in the effective treatment and prevention of influenza. Oseltamivir and zanamivir are both approved by the Food and Drug Administration (FDA) for use in controlling both influenza A and B viruses (*1*). Oseltamivir phosphate is the active ingredient in Tamiflu (F. Hoffmann-La Roche Ltd., Basel, Switzerland) and is available in capsule and powder form.

The specter of an avian influenza pandemic has given Tamiflu much notoriety, and anticipation of the potential public health threat has prompted a demand for the product. Consequently, criminal elements have already begun to produce counterfeit Tamiflu. Recently, US Customs agents seized counterfeit Tamiflu entering the United States (*2*). The bogus products, which contained vitamin C and lacked the active ingredient of oseltamivir phosphate, had been purchased through the Internet. Although these shipments were quickly detected by a joint effort of the FDA and US Customs and Border Protection, these products would easily have gone unnoticed in developing countries, where insufficient resources and infrastructure hamper the ability to monitor and preserve drug quality. WHO estimates that up to 25% of the medicines consumed in developing countries are counterfeit or substandard (*3*).

Simple and affordable colorimetric assays provide a practical means to rapidly monitor drug quality in resource-poor areas. Because oseltamivir phosphate (Figure 1) possesses amine groups, the protonated form may act as a cationic site for anionic dyes such as Congo red and bromochlorophenol blue to produce colored ion-pairing complexes. Congo red has been used in colorimetric determinations of chitosan and poly (*N*-vinyl-2-pyrrolidone) while bromophenol blue has been used in colorimetric assays for antimalarial drugs (*4**-**6*). Therefore, our objective was to develop and evaluate a colorimetric technique, as well as a high-performance liquid chromatographic method (HPLC), to measure the concentration of oseltamivir phosphate in pharmaceutical preparations. The HPLC method described here was used to validate the colorimetric test. To date, there are few published reports of HPLC methods for measuring oseltamivir. A sensitive HPLC-mass spectrometry assay for oseltamivir carboxlate in plasma and urine and an HPLC assay for oseltamivir phosphate in pharmaceutical preparations have been described (*7**,**8*). In our study, we validated, compared, and applied colorimetric and HPLC techniques to the testing of alleged Tamiflu product purchased through the Internet.

**Figure 1:**
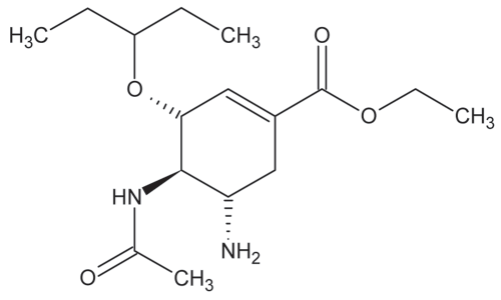
Structure of oseltamivir.

## Methods

### Reagents and Apparatus

All reagents were of analytical-reagent grade, and deionized water was used for all aqueous solutions. Pharmaceutical-grade oseltamivir phosphate was graciously donated by Hoffman-La Roche Ltd. We purchased Congo red (dye content ≈97%), bromochlorophenol blue sodium salt (dye content ≈95%), potassium hydrogen phthalate, monobasic potassium phosphate, sodium bicarbonate, and sodium hydroxide from Sigma-Aldrich (St. Louis, MO, USA); HPLC-grade acetonitrile from Mallinckrodt Baker, Inc. (Phillipsburg, NJ, USA); and ethyl acetate from Acros Organics (Morris Plains, NJ, USA).

Absorbance measurements were taken using a Spectronic 21 spectrophotometer (Milton Roy, Riviera Beach, FL, USA). HPLC analysis was conducted with an Agilent 1100 Series system (Agilent, Palo Alto, CA, USA) using an X-Terra, RP18, 4.6- × 150-mm column (Waters, Milford, MA, USA).

### Sample Preparation

The colorimetric and HPLC methods were evaluated in terms of linearity, assay precision, and accuracy by using pharmaceutical preparations compounded from a mixture of lactose, starch, talc, povidone K30, croscarmellose, and stearyl fumarate, which contained known amounts of oseltamivir phosphate. These inactive ingredients (excipients) are those found in the capsule formulation of Tamiflu (*9*). The excipient mix was kept constant while various ratios of oseltamivir phosphate and lactose were added to produce sample groups containing 0%, 50%, 80%, 100%, 120%, and 150% of the amount of active ingredient normally found in a Tamiflu capsule. The 100% mixture contains 46% of the active ingredient and is equivalent to a capsule containing 75 mg of oseltamivir base (98.5 mg oseltamivir phosphate).

We conducted a search for Tamiflu products on the Internet using the keywords “Tamiflu,” “prescription,” and “cheap or inexpensive.” Approximately 40 online sources were compiled and sorted according to price. We acquired the 6 cheapest products that did not require a prescription and tested for active ingredient by using both colorimetric methods and HPLC. All the products were in capsule form and allegedly contained 75 mg of oseltamivir base (98.5 mg oseltamivir phosphate) as described in the package insert. The contents of the entire capsule were deposited into a glass vial, and 32.8 mL of water (Congo red test) or 8.2 mL (bromochlorophenol blue test) of water was added. The mixture was vigorously shaken for ≈10 s, allowed to equilibrate for 10 min, shaken again, and then filtered through 0.22- or 0.45-μm membranes. The amount of oseltamivir per capsule was then determined by using the colorimetric and HPLC methods.

### Colorimetric Assay

Congo red and bromochlorophenol blue salt were evaluated for the colorimetric assay and prepared at a concentration of 1 mg/mL in water. For the Congo red test, a portion of material from each sample group was weighed, and enough water was added to achieve a concentration of 6.5 mg/mL. This is equivalent to 3 mg/mL of oseltamivir phosphate present in the 100% sample group. The filtered sample solution (0.150 mL) was added to a glass siliconized tube containing 0.250 mL of Congo red solution, 0.350 mL of 0.1 M phthalate buffer, pH 4.2, and 3 mL of ethyl acetate. The tubes were capped and the mixture vigorously shaken for 10 s. After complete phase separation, the top organic layer (red, if oseltamivir was present) was transferred to a 13-mm diameter clean glass tube for absorbance measurements at 520 nm. For the bromochlorophenol blue test, the sample was prepared so that the final concentration for the 100% group was 26 mg/mL, which is equivalent to 12 mg/mL of oseltamivir phosphate. The sample was mixed and filtered as described previously, and 0.150 mL was added to a siliconized glass tube containing 0.250 mL of bromochlorophenol blue solution, 0.350 mL of 0.1 M phosphate buffer, pH 7.0, and 3 mL of ethyl acetate. After vigorous mixing and phase separation, the top organic layer (blue, if oseltamivir was present) was transferred to a 13-mm diameter clean glass tube for absorbance measurements at 590 nm. Other drugs commonly used in developing countries, i.e. aspirin, ampicillin, chloroquine, acetaminophen, amoxicillin, ciprofloxacin, quinine, chloramphenicol and erythromycin, were prepared in water at a concentration of 2.5 mg/mL and tested using the described colorimetric conditions.

### HPLC Analysis

We used a mobile phase comprising 30% acetonitrile and 70% 0.05 M bicarbonate buffer, pH 10, at a flow rate of 1 mL/min to achieve component separation while maintaining column temperature at 30^o^C. Oseltamivir was detected by UV absorbance at 254 and 220 nm with a retention time of ≈4 min. Injection volume was 2 μL. The limit of detection was determined from the analyte mass equivalent to 3 times the baseline noise.

## Results and Discussion

### Colorimetric Assay

**Figure 2:**
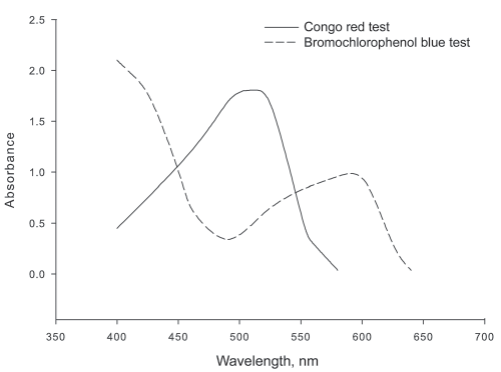
Spectra of Congo red and bromochlorophenol blue complexes with oseltamivir in ethyl acetate.

Optimal formation of the complex is dependent on the ionization constants (pKa) as well as solubility characteristics for both the basic drug and acidic dye; therefore, optimum complex formation is pH dependent and is characteristic of the analyte being tested. We determined the optimum pH for complex formation between oseltamivir and Congo red to be 4; the optimum pH for bromochlorophenol blue and oseltamivir was 6–7. The absorption spectra for the oseltamivir–Congo red complex (maxima 507 nm) and oseltamivir-bromochlorophenol blue complex (maxima 589 nm) are shown in Figure 2. We evaluated selectivity of the Congo red assay with other commonly used pharmaceuticals. Under the described assay conditions, aspirin, ampicillin, chloroquine, acetaminophen, amoxicillin, ciprofloxacin, and chloramphenicol produced a clear colorless organic phase; quinine and erythromycin showed a very faint rose color. Of the drugs tested for specificity using the bromochlorophenol blue assay, quinine produced a purple color, chloroquine a light blue color, and acetaminophen a faint yellow color. The selectivity of the assay is a function of drug solubility in water as well as pH. Oseltamivir phosphate is highly soluble in water (*9*). Because aspirin, acetaminophen, amoxicillin, quinine, chloramphenicol, and erythromycin are insoluble or slightly soluble in water, most of the material was eliminated by filtration before the assay was conducted and may have contributed to a colorless ethyl acetate phase. Therefore, filtration is considered necessary because aqueous solubility and a pKa confer selectivity of the colorimetric tests with oseltamivir.

Evaluation of the colorimetric assay for oseltamivir carboxylate, the active metabolite of the prodrug oseltamivir phosphate (*9*), has not been performed. Ion-pairing with acidic dyes under the described conditions is less likely because the carboxy metabolite is a zwitterion.

**Figure 3:**
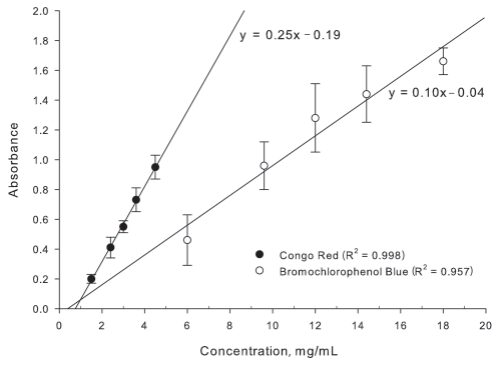
Linearity of colorimetric assays.

Table 1 shows the intraday and interday variability associated with the colorimetric assays; Figure 3 illustrates the linearity of the absorbance versus concentration curve. Greater linearity and lesser variability are observed from the Congo red assay. Note that the variability also includes deviations arising from the preparation of the oseltamivir formulations. The greater slope associated with the Congo red curve relative to the bromochlorophenol blue curve in Figure 3 demonstrates a more sensitive assay. The results of the colorimetric assays for the oseltamivir phosphate products purchased over the Internet are shown in Table 2 and compared with values determined from HPLC analysis. These values are the percentage of active ingredient found per capsule relative to that stated on the manufacturer’s package insert. All, except brand B (Cipla), were Roche brand products. The senders’ addresses for brands C, D, and E were all within the United States, while brand C originated from India and brands A and F originated from Greece. Except for brand B, all products were within ±10% of the stated amount of active ingredient.

**Table 1:** Accuracy and precision for the high-performance liquid chromatographic (HPLC) and colorimetric assays (n = 5)

Nominal concentration, mg/mL	Accuracy, %			Precision, %	
	Interday	Intraday		Interday	Intraday
HPLC					
0.6	–10.8	–10.3		9.0	7.9
1.5	–8.4	0.4		7.3	2.3
2.4	5.0	–0.7		7.3	5.7
3.0	0.4	1.6		4.9	4.8
3.6	0.7	4.4		4.1	3.6
4.5	–0.9	–3.2		2.5	2.5
Congo red colorimetric					
1.5	2.0	2.2		12.5	2.3
2.4	2.6	–0.3		9.2	6.9
3.0	–5.5	1.0		6.5	5.4
3.6	0.7	1.2		3.7	3.8
4.5	1.0	–0.1		2.6	1.3
Bromochlorophenol blue colorimetric					
6.0	–5.3	–19.4		18.6	12.4
9.6	0.0	3.4		13.5	2.6
12.0	2.7	10.4		6.1	5.9
14.4	3.5	3.2		4.3	3.8
18.0	–2.9	–5.4		4.9	3.0

**Table 2:** Evaluation of oseltamivir products purchased over the Internet*

Brand	HPLC	Congo red	Bromochlorophenol blue
A	94 ± 1	95 ± 3	106 ± 4
B	87 ± 2	88 ± 3	97 ± 8
C	94 ± 4	93 ± 2	103 ± 3
D	96 ± 1	93 ± 5	107 ± 7
E	97 ± 3	89 ± 3	104 ± 5
F	95 ± 0	88 ± 2	101 ± 3

*HPLC, high-performance liquid chromatography. Values are the percentage of active ingredient found per capsule relative to that stated on the manufacturer’s package insert (average ±SD; n = 3 capsules).

### HPLC Analysis

**Figure 4:**
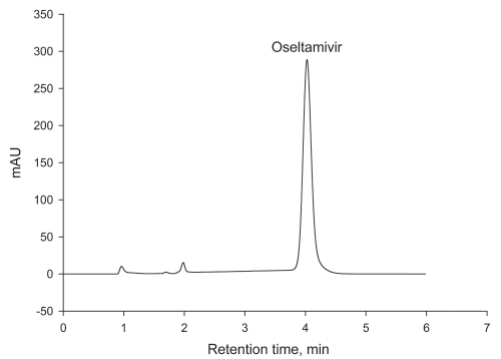
Chromatogram of oseltamivir from Tamiflu purchased over the Internet.

Intraday and interday accuracy and precision were within ±11% for oseltamivir phosphate concentrations of 0.6 mg/mL to 4.5 mg/mL (Table 1). Mobile phase pH above the pKa of a basic analyte generally produces chromatograms with a good symmetrical peak shape. The chromatogram for oseltamivir is shown in Figure 4. Because the pKa of oseltamivir is 7.75 (*9*), a mobile phase comprising a pH 10 (2 U above the pKa) bicarbonate buffer was chosen. The C18 column used for the HPLC method is designed to operate under basic pH conditions. Injections of aqueous mixtures of aspirin, ampicillin, chloroquine, acetaminophen, amoxicillin, ciprofloxacin, quinine, chloramphenicol, or erythromycin into the HPLC system showed no interfering chromatographic peaks. The limit of detection for oseltamivir phosphate at 220-nm and 254-nm detection wavelengths are 2.2 ng and 4.2 ng, respectively.

## Conclusions

Anionic dyes such as Congo red and bromochlorophenol blue form colored ion-pairing complexes with oseltamivir to produce a colored product extractable in ethyl acetate. The Congo red method produces a colored product, which is more linearly proportional to oseltamivir concentration, has less variability, and is more selective than the bromochlorophenol blue method.

Colorimetric tests are rapid and easy to perform. The reagents and equipment for colorimetric tests are inexpensive, relatively nontoxic, and are ideal for use in field situations.

## References

[1] Centers for Disease Control and Prevention. Influenza antiviral medications: 2000–06 chemoprophylaxis (prevention) and treatment guidelines [cited 2006 Aug 21]. Available from http://www.cdc.gov/flu/professionals/treatment/0506antiviralguide.htm

[2] US Customs and Border Protection. San Francisco Customs and Border Protection officers seize counterfeit Tamiflu. Press release. 2005 Dec 19 [cited 2006 Aug 21]. Available from http://cbp.customs.gov/xp/cgov/newsroom/news_releases/archives/2005_press_releases/122005/12192005.xml.

[3] World Health Organization. Fact sheet 275. Counterfeit medicines. November 2006 [cited 2008 Jan 25]. Available from http://www.who.int/mediacentre/factsheets/fs275/en/index.html

[4] Muzzarelli RA. Colorimetric determination of chitosan. Anal Biochem. 1998;260:255–7. 10.1006/abio.1998.2705 9657888

[5] Riedhammer TM. Colorimetric determination of poly(N-vinyl-2-pyrrolidone) in contact lens solutions. J Assoc Off Anal Chem. 1979;62:52–5. 422505

[6] El-Ashry SM, Aly FA, El-Brashy AM. Studies of complex formation between the bromophenol blue and some important aminoquinoline antimalarials. Arch Pharm Res. 1994;17:415–9. 10319150 10.1007/BF02979117

[7] Wiltshire H, Wiltshire B, Citron A, Clarke T, Serpe C, Gray D, Development of a high-performance liquid chromatographic-mass spectrometric assay for the specific and sensitive quantification of Ro 64–0802, an anti-influenza drug, and its pro-drug, oseltamivir, in human and animal plasma and urine. J Chromatogr B Analyt Technol Biomed Life Sci. 2000;745:373–88. 10.1016/S0378-4347(00)00300-5 11043756

[8] Lindegardh N, Hien TT, Farrar J, Singhasivanon P, White NJ, Day NPJ. A simple and rapid liquid chromatographic assay for the evaluation of potentially counterfeit Tamiflu®. J Pharm Biomed Anal. 2006;42:430–3. 10.1016/j.jpba.2006.04.028 16750606

[9] F. Hoffman-La Roche Ltd. Product monograph. Tamiflu [cited 2008 Jan 25]. Available from http://www.rochecanada.com/gear/glossary/servlet/staticfilesServlet?type=data&communityId=re753001&id=static/attachedfile/re7300002/re77300002/AttachedFile_07547.pdf

